# Targeting the molecular chaperone SlyD to inhibit bacterial growth with a small molecule

**DOI:** 10.1038/srep42141

**Published:** 2017-02-08

**Authors:** Amit Kumar, Jochen Balbach

**Affiliations:** 1 Astbury Centre for Structural Molecular Biology, School of Molecular and Cellular Biology, University of Leeds, Leeds LS2 9JT, UK; 2 Institute of Physics, Biophysics, Martin Luther University, Halle, Wittenberg, Germany; 3 Centre for Structure und Dynamics of Proteins (MZP), Martin Luther University Halle, Wittenberg, Germany

## Abstract

Molecular chaperones are essential molecules for cell growth, whereby they maintain protein homeostasis. Because of their central cellular function, bacterial chaperones might be potential candidates for drug targets. Antimicrobial resistance is currently one of the greatest threats to human health, with gram-negative bacteria being of major concern. We found that a Cu^2+^ complex readily crosses the bacterial cell wall and inhibits SlyD, which is a molecular chaperone, *cis*/*trans* peptidyl prolyl isomerise (PPIase) and involved in various other metabolic pathways. The Cu^2+^ complex binds to the active sites of SlyD, which suppresses its PPIase and chaperone activities. Significant cell growth retardation could be observed for pathogenic bacteria (e.g., *Staphylococcus aureus* and *Pseudomonas aeruginosa*). We anticipate that rational development of drugs targeting molecular chaperones might help in future control of pathogenic bacterial growth, in an era of rapidly increasing antibiotic resistance.

Antibiotics are the drugs of choice to treat bacterial infections. Because of the emergence of dangerous multi-drug resistant strains of bacteria, currently approved antibiotics are losing base against bacterial diseases^1^,^2^. Recent studies suggest that by 2050 a continuous rise in antibacterial resistance might result in the death of 10 million people per year^3^. Among pathogenic bacteria, ‘ESKAPE pathogens’ are of particular concern. Within this class of organisms, ‘KAPE’ represent gram-negative bacteria with increased susceptibility to attain multi-drug resistance^4^. Gram-negative bacteria are more threatening to humans due to the presence of the outer polysaccharide layer and multi-drug efflux transporters. This renders many small molecules including antibiotics eventually ineffective. This phenomenon has led to the creation of novel antibacterial agents distinct from the existing classes of molecules and their targets as well. A present interest is focused towards water soluble metal-based small molecular systems with organic moieties, which can cross the cell wall barrier and specifically target crucial bacterial molecules. Attention has been paid to coordination complexes because of their antimicrobial^5^,^6^, antifungal^7^,^8^,^9^ and anticancer activities^10^,^11^ applied in industry, ecology, or medicine. These complexes exhibited versatile electrochemical properties with tunable coordination chemistry which subsequently offer an enormous scope for the design of new entities. Cis-platin and its derivatives are well known examples which interact with DNA and human superoxide dismutase accounting for leads as both an antitumor drug and to fight amyotrophic lateral sclerosis^12^,^13^. Cell toxicity is well known for copper itself however its complexes often exhibit several fold better responses^10^,^14^,^15^,^16^. We previously showed the anticancer activity of copper(II) complexes exhibiting up to 100 fold better responses compared to Cu^2+ ^10,14.

One of the important functions of protein-folding helper proteins is to interact with aggregation-prone protein folding intermediates by recognizing non-natively exposed hydrophobic patches and shuffling disulfide bond (disulfide isomerases) or to accelerate peptidyl–prolyl *cis–trans* isomerization^17^,^18^. SlyD harbours both a peptidyl–prolyl isomerase and chaperone activity^19^,^20^ and is involved in several bacterial metabolic pathways including twin-arginine translocation (Tat) transport, biosynthesis of [NiFe] hydrogenases, and metal storage/release^21^. SlyD and its homologous proteins are present in all prokaryotes and archaea^21^,^22^. SlyD had first been characterized because of its PPIase and molecular chaperone activities^23^, which are located on two separate domains but mechanistically coupled^21^,^24^,^25^,^26^,^27^. Additionally, deletion of the slyD gene resulted in a marked reduction of the hydrogenase activity in cell extracts prepared from anaerobic *Escherichia coli* cultures^28^ and the slyD gene was classified as ‘Death Gene’ because it is involved in a pathway that leads to death of a majority of cells^29^. Moreover, being unique to prokaryotes and archaea; and essential for organism survival, these molecules could become interesting targets for developing small molecules which obstruct their activity inside the cells.

For several coordination compounds, antiprolifirative, antimicrobial, and antifungal activity had been identified by co-incubating them with cell cultures. However, their molecular mechanisms^30^,^31^,^32^ of cell death often remain elusive^33^. In structure based drug-design, to a large extent the proposed complexes hardly cross the cell wall/membrane barrier to reach their targets in the complex cellular environment, although they are quite effective binding-partners under *in vitro* conditions^33^. In our present studies, a coordination complex of Cu^2+^ and anthracenyl terpyridine (Fig. 1a) (hereafter refer as Cu^2+^ complex)^10^ has been found to inhibit bacterial growth by blocking the active side of SlyD (present in prokaryotes and archaea). We observed by *in cell* NMR that this promising antibacterial activity concomitantly occurs with Cu^2+^ complex binding to the SlyD. *In vitro* NMR spectroscopy showed that Cu^2+^ complex interacts with most of the residues forming the PPIase active side of the FKBP domain. These interactions inhibit both chaperone and PPIase activity of SlyD. We propose that these missing functions of SlyD led to a negative control of major metabolic pathways and hence a retardation of cell growth of *E. coli, S. aureus* (gram-positive) and *P. aeruginosa* (gram-negative) was observed.

## Results

### The Cu^2+^ complex inhibits bacterial growth

Metal ions cannot easily pass the cell wall of gram-negative bacteria such as *E. coli*^34^. Despite their complex composition (lipopolysaccharides, lipoproteins and peptidoglycans), however, the Cu^2+^ complex is able to enter inside of the *E. coli* (Figure S1). To evaluate the antibacterial property, growth curves were analyzed up to the stationary phase for the *E. coli* strain BL21(DE3). Complete bacterial growth inhibition was observed by incubation above a concentration of 2 μM of the Cu^2+^ complex. Lower concentrations (1.0 μM and 0.5 μM) inhibited only about 70–80% while beyond these concentrations cell growth was not effective (Figure S2).

### Role of SlyD during cell growth inhibition

**Figure 1:**
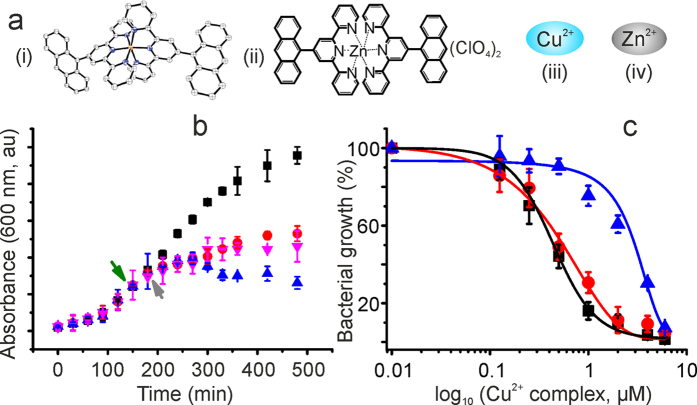
Molecular systems and their effect on bacterial growth. (**a**) Small molecules used in the present study: (i) Cu^2+^ complex, (ii) Zn^2+^ complex, (iii) Cu^2+^ and (iv) Zn^2+^. (**b**) Growth curve analysis showing the role of SlyD in survival of bacteria against Cu^2+^ complex. Time dependent optical density of *E. coli* BL21(DE3) transformed with *Ec*SlyD. The green arrow indicates the time point of IPTG induction (■ and 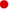 curve) or addition of Cu^2+^ complex (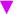 curve) and grey arrow indicated the addition of the Cu^2+^ complex (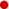 and 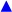 curve) or IPTG (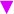 curve). (c) IC_50_ value determination for *E. coli* strain BL21(DE3): ■–*E. coli* strain BL21(DE3), 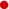
–cells transformed with *Ec*SlyD (non-induced), and 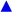
–cells transformed with *Ec*SlyD and induced with IPTG. In (**b**) and (**c**), error bars represent mean ± s.d. for triplicate experiments.

To evaluate the role of the molecular chaperone SlyD in the growth and maintenance of bacterial cells after treatment with the Cu^2+^ complex, growth curves were analyzed with cells over expressing *E. coli* SlyD (1–165) (*Ec*SlyD). At an optical density of 0.5–0.6 of exponentially growing *Ec*SlyD gene transformed *E. coli* BL21(DE3) cells (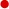 and ■ symbols in Fig. 1b), SlyD over expression was induced by IPTG (green arrow in Fig. 1b). After 30 minutes of IPTG induction, cultures (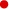 and 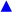) were supplemented with 4 μM of the Cu^2+^ complex (grey arrow in Fig. 1b). After 2 h, no further increase in absorbance was observed in the non-induced bacteria (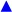 in Fig. 1b). However, the IPTG-induced bacterial cells showed a significant survival against the Cu^2+^ complex induced toxicity (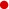 in Fig. 1b). The latter cells could not grow as normal as the non-treated control due to the inhibitory role of the Cu^2+^ complex at 4 μM which could not be compensated by the presence of a high numbers of *Ec*SlyD molecules. To show further the involvement of SlyD in growth and inhibition, the *Ec*SlyD gene transformed *E. coli* BL21(DE3) cells were initially grown up to optical densities of 0.5–0.6 and then 4 μM of Cu complex was added (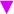, indicated by green arrow) and further grown for 30 minutes. Thereafter the cells were induced with IPTG (indicated by the grey arrow). These cells showed better survival after Cu^2+^ complex treatment. Note that non-induced bacteria showed a similar growth curve as *E. coli* strain BL21(DE3) without *Ec*SlyD transformation (Figure S3).

The *in vitro* antimicrobial activity of the Cu^2+^ complex in terms of half-maximal inhibitory concentration (IC_50_)^31^ was found to be 0.44 ± 0.02 μM and 0.55 ± 0.01 μM for *E. coli* strain BL21(DE3) and *E. coli* strain BL21(DE3) containing the *Ec*SlyD plasmid but non-induced, respectively (Fig. 1c). These studies suggested the potential of the Cu^2+^ complex in inhibiting the bacterial growth. For the *Ec*SlyD over expressing *E. coli* strain BL21(DE3) an IC_50_ value of 2.89 ± 0.09 μM was measured (Fig. 1c). This ~5 fold increased IC_50_ is in line with the increase in the optical density observed in the respective growth curves (Fig. 1b). Note, that free Cu^2+^ did not show significant bacterial inhibition at the concentrations studied herein (Figure S4). As a second control, we employed 9-anthracene methanol in the absence of Cu^2+^, which showed only 10–20% of inhibition at maximum concentration. Third, we replaced Cu^2+^ with Zn^2+^ in the coordination complex^10^,^35^. This molecule showed growth inhibition (10–20%) similar to 9-anthracene methanol (Figure S4). Together, these control experiments confirm that a Cu-metal centre in the 9-anthracene-terpyridine complex is required for efficient growth inhibitor.

### Interactions of the Cu^2+^ complex with *Ec*SlyD studied by *in cell* NMR

**Figure 2:**
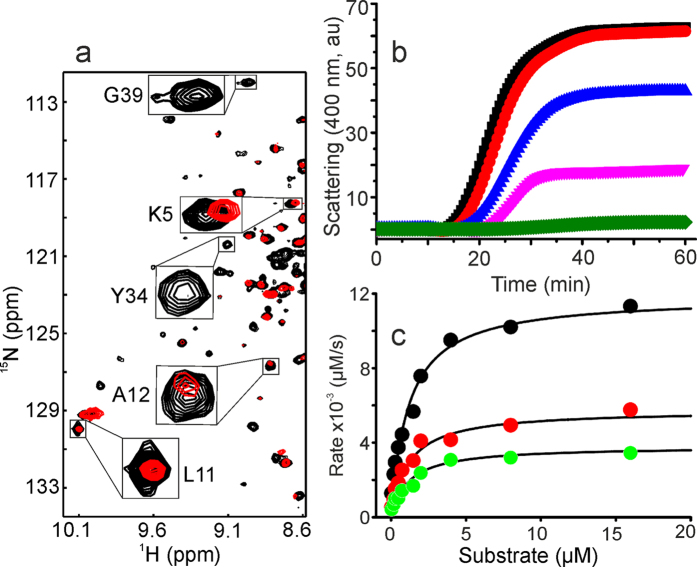
(**a**) *In cell* NMR revealing the binding of the Cu^2+^ complex to *Ec*SlyD inside BL21(DE3) cells. Overlaid sections of 2D ^1^H-^15^N HSQC spectra are shown before (black) and after addition of the Cu^2+^ complex. Enlarged boxes indicate examples of cross peaks showing decreased intensity, changes in chemical shifts and missing cross peaks upon Cu^2+^ complex interaction. (**b**) Inhibition of molecular chaperone activity of *Ec*SlyD by Cu^2+^ complex. Symbols indicated the insulin aggregation. Conditions: ■–control experiment without *Ec*SlyD, 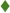
–in the presence of 40 μM *Ec*SlyD; 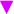, 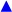 and 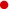
–inhibition of chaperone activity by Cu^2+^ complex at 40, 70 and 100 μM, respectively. (**c**) Inhibition of the PPIase activity of *Ec*SlyD. PPIase activity was carried out at 25 μM (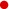) and 50 μM (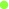) of Cu^2+^ complex taking no inhibitor as a control (●). The data points were fitted according to the Michaelis-Menten equation.

*In cell* NMR spectroscopy is a well suited method to investigate the conformation, stability and dynamics of proteins inside living cells^36^,^37^,^38^,^39^. The high complexity of the intracellular environment makes it difficult to analyze the interactions of small molecules with the protein of interest under physiological conditions due to low protein concentrations and a high background for NMR experiments. To investigate the interaction of *Ec*SlyD with the Cu^2+^ complex inside *E. coli* BL21(DE3) cells, we recorded 2D ^1^H-^15^N HSQC spectra after IPTG induction. The induction level is high enough to detect the well-dispersed resonances of *Ec*SlyD (black contours in Fig. 2a). Between 7.5 ppm and 8.5 ppm, backbone amide protons are overlapped by cellular background signals (Figure S5a). After incubation with the Cu^2+^ complex, several resonances either changed their chemical shifts or showed decreased intensities. These spectral changes confirm that the Cu^2+^ complex indeed can pass the cell wall of *E. coli* and interact with the molecular chaperone *Ec*SlyD. To exclude that *Ec*SlyD from outside the cells contributes to the spectra e.g. due to cell leakage, we recorded a 2D ^1^H-^15^N HSQC of the supernatant indicating no detectable signals (Figure S5b).

### Inhibition of the molecular chaperone activity of *Ec*SlyD

Next, we examined, whether the *in cell* NMR detected binding of the Cu^2+^ complex to *Ec*SlyD relates to its chaperone and PPIase activity. One established assay for molecular chaperone activity is the suppression of aggregation of the insulin B chain after reduction by DTT (black and green symbols in Fig. 2b)^26^,^40^. This chaperone function could be inhibited by stoichiometric amounts of *Ec*SlyD in a concentration-dependent fashion shown in Fig. 2b. This observation clearly suggests that the chaperone activity of SlyD can be controlled by the Cu^2+^ complex. Note, Cu^2+^ perchlorate, 9-anthracene methanol or the corresponding Zn^2+^ complex showed a negligible effect on inhibition of insulin aggregation under these conditions (Figure S6).

### Inhibition of *cis*/*trans* peptide prolyl isomerase (PPIase) activity

The residues responsible for PPIase activity in SlyD reside in the FKBP domain^26^,^41^. The catalytic PPIase activity of *Ec*SlyD was examined with a standard assay, which is the catalysis of refolding of the reduced and carboxymethylated form of the S54G/P55N variant of ribonuclease T1 (RCM-T1), which is limited by a slow *trans* to *cis* isomerization reaction of Pro39^22^. *Ec*SlyD showed a *K*_m_ value of 1.4 ± 0.4 μM (black symbols in Fig. 2c) which is in good agreement with the literature value^22^. In the presence of 25 μM and 50 μM of the Cu^2+^ complex this PPIase assay suggests non-competitive inhibition of *Ec*SlyD as judged by the corresponding Michaelis–Menten plots (Fig. 2c). Data analysis yields a *K*_i_ value of 23.6 μM.

### Binding of the Cu^2+^ complex to *Ec*SlyD by fluorescence spectroscopy

**Figure 3:**
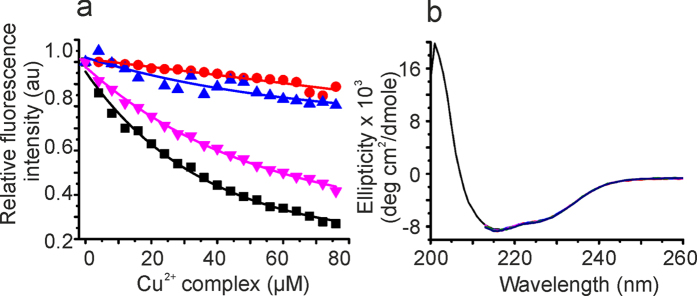
Binding studies of the Cu^2+^ complex with EcSlyD. (**a**) Relative fluorescence intensity plots of *Ec*SlyD analysed at 335 nm during titration with ■–Cu^2+^ complex, 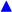
–Cu^2+^. 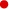
–9-anthracene methanol and –∆IF SlyD *vs* Cu^2+^ complex. (**b**) CD spectrum of *Ec*SlyD in the presence and absence of Cu^2+^ complex (10 μM to 50 μM). The black spectrum corresponds to free *Ec*SlyD. High absorption beyond 215 nm did not allow useful spectra to be obtained upon addition of the Cu^2+^ complex.

To gain insights into the molecular details of the *Ec*SlyD inhibition, we performed a fluorescence titration experiment with the Cu^2+^ complex (Fig. 3a). The intrinsic *Ec*SlyD fluorescence intensity dominated by Tyr 34 and Tyr 68 in the FKBP domain and Tyr 71 in the linker between FKBP and IF domain. Intensity dropped as a function of the Cu^2+^ complex concentration, resulting in a dissociation constant (*K*_D_) of 45 μM. We found a 2:1 stoichiometry and assumed the same affinity for two binding sites at *Ec*SlyD. In contrast, Cu^2+^ or 9-anthracene methanol did not result in a significant decrease in the fluorescence intensity (Fig. 3a). Additionally, we studied ∆IF *Ec*SlyD, which showed a *K*_D_ of 63.65 ± 4.5 μM for the Cu^2+^ complex, indicating that the IF domain only marginally modifies the affinity to the inhibitor. Neither the Cu^2+^ complex nor the latter two compounds changed the far UV-CD spectrum of *Ec*SlyD indicating that the structural integrity of the protein remains intact (Fig. 3b).

### Residue specific interaction of the Cu^2+^ complex with *Ec*SlyD

**Figure 4:**
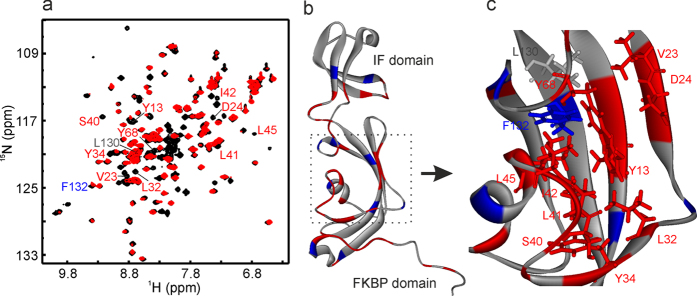
*In vitro* NMR spectroscopic analysis of the Cu^2+^ complex binding to EcSlyD. (**a**) 2D ^1^H-^15^N HSQC overlaid spectra of ^15^N *Ec*SlyD in the free (black) and bound state (red). Red and blue labels are the residues involved in the PPIase activity. (**b**) Mapping of cross peak intensities on *Ec*SlyD structure. Residues with missing NMR resonances are marked in red, 20–50% loss in intensities are indicated in blue and no significant change in grey. (**c**) Close up view of the PPIase active sites. Key residues are labeled by their side chains using the same color coding as in (**b**).

*In vitro* NMR spectroscopy^42^,^43^ can be applied to probe the binding of the Cu^2+^ complex to *Ec*SlyD at residue-level resolution. The applied 2D ^1^H−^15^N-HSQC experiment solely detects nitrogen-bound protons of ^15^N-*Ec*SlyD. At a stoichiometry of 1:2 of *Ec*SlyD versus the Cu^2+^ complex we analysed differences in the chemical shifts and resonance intensities of individual residues. Several resonances were broadened beyond the detection limits, while other residues changed their chemical shift positions (Fig. 4a). Mapping these properties onto the structure shows that significant changes are at the FKBP domain and few at the IF domain (Fig. 4b). *Ec*SlyD residues Y13, V23, D24, L32, Y34, S40, L41, I42, L45, Y68, L130, and F132 are important for the PPIase activity of the FKBP domain^26^. Interestingly, the intensity of all of these residues decreased upon addition of the Cu^2+^ complex (Fig. 4c) with only one exception (L130). This confirms the observed inhibition of PPIase activity by the Cu^2+^ complex, because it binds to the active site of *Ec*SlyD. Similarly, the residues of the IF domain were analysed. Upon Cu^2+^ complex addition the intensities of Leu75, Val76, Thr100, Gln102, Gly103, Val105, and Gly121 were strongly affected, while those of Ala98 and Val119 were moderately affected (Fig. 4b). These results are in good agreement with the inhibition of the chaperone activity by the Cu^2+^ complex.

In order to study the binding affinity of the Cu^2+^ complex to the individual *Ec*SlyD domains, the NMR intensities of titrating residues were analysed (Figure S9). Residues of the FKBP domain yielded an averaged *K*_D_ value of 87 ± 30 μM and for the IF domain we found 105 ± 31 μM. The values match well with the affinity obtained with fluorescence spectroscopy. Cu^2+^ perchlorate and 9-anthracene methanol did not show interactions with these residues of the FKBP and IF domain corresponding to the respective PPIase and chaperone function (Figure S8).

### Toxicity of the Cu^2+^ complex for pathogenic bacterium

**Figure 5:**
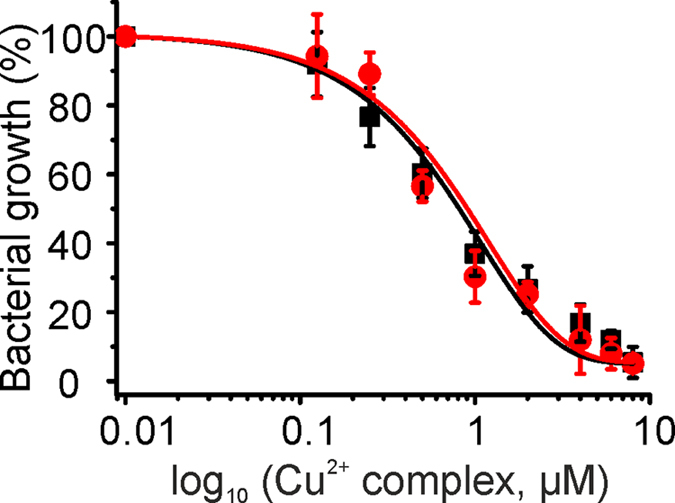
Growth analysis of pathogenic bacteria in presence of the Cu^2+^ complex. IC_50_ value were determined for *Staphylococcus aureus* (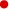) and *Pseudomonas aeruginosa* (■). Error bars represent mean ± s.d. for triplicate experiments.

We tested the toxicity of the Cu^2+^ complex against the pathogenic bacteria *Staphylococcus aureus* (gram-positive) and *Pseudomonas aeruginosa* (gram-negative) as chosen from the ‘ESKAPE pathogens’ category^4^. The IC_50_ was found to be 0.90 ± 0.1 μM and 0.81 ± 0.07 μM for *Staphylococcus aureus* and *Pseudomonas aeruginosa*, respectively (Fig. 5). The results indicate that the Cu^2+^ complex is effective even for pathogenic bacteria. The obtained IC_50_ values are close to the findings with *E. coli*. Note again, that Cu^2+^ perchlorate, 9-anthracene methanol, and the Zn^2+^ complex have a negligible effect on bacterial growth (Figure S10).

## Discussion

Molecular chaperones are key molecules for cell growth and maintenance^44^. SlyD is a metallo-chaperone^21^ in prokaryotes and archaea additionally harbouring a PPIase domain^22^ and a C-terminal Ni^2+^ binding domain to deliver these metal ions during [NiFe]-hydrogenase maturation^45^. Furthermore, SlyD guides proteins to the Tat-dependent translocation of folded proteins across the cytosolic membrane of bacteria^46^. Targeting such crucial functions to inhibit bacterial growth might be a new strategy to counteract the emergence of multi-drug resistance. Coordination complexes are gaining interest in this respect for medical applications^11^,^47^. Although known coordination complexes exhibit promising antibacterial activities, their mechanisms of action are less explored. Recent examples include the complexes of [CuX_2_(INH)_2_]·nH_2_O (X = Cl and n = 1; X = NCS and n = 5; X = NCO and n = 4; INH = isoniazid), which have been optimized for *in vitro* antibacterial activity^48^. The compound N-(2-hydroxybenzylidene)-1-ethyl-1, 4-dihydro-7-methyl-4-oxo-1, and 8 naphthyridine-3-carbohydrazide and their Cu(II), Co(II) and Zn(II) complexes were synthesized and characterized and their antibacterial activities have been tested under *in vitro* conditions^49^. Cu(II) complexes with the chemotherapeutic antibacterial norfloxacin and N-donor mixed-ligands have been recently reported^50^.

For the Cu^2+^ complex studied here, we found at low micromolar concentrations antibacterial activity after readily crossing the bacterial cell wall probably because of the hydrophobic character of the anthracenyl terpyidine moiety. *In vivo* NMR revealed that *Ec*SlyD is involved in this inhibition (Fig. 1). Overexpression of *Ec*SlyD cannot fully overcome the inhibitory function of the Cu^2+^ complex indicating that this complex might influence additional cellular processes (Fig. 1 and Figures S2 and S3).

Few molecular chaperones such as heat shock proteins (HSPs) have been proposed as drug targets in cancer therapy^51^. More recently, Kondoh and Osada performed high throughput screens to identify molecular chaperones for the same purpose^52^. Geldanamycin and radicicol have been identified as inhibitors of HSP90^53^. Other chaperones such as HSP60 or HSP70 have received much less attention as a potential drug target^51^. To the best of our knowledge bacterial chaperones have not been reported in this context so far. It is well known that antibacterial small molecules can interfere with cell wall synthesis (e.g., penicllins), cell membrane function (e.g., polymixin B), protein synthesis (e.g., aminoglycosides) and nucleic acid synthesis (e.g., quinolones)^54^.

The here presented Cu^2+^ complex readily crosses the bacterial cell wall which might be of advantage in terms of drug resistance e.g. by efflux-mediated extrusion. Nano-particles have recently emerged in this area^55^,^56^,^57^ because of their slightly larger diameters compared to the drug efflux pumps^58^,^59^. Vancomycin-capped gold nano-particles enhanced the *in vitro* antibacterial activities of vancomycin-resistant *Enterococcus* and *E. coli* strains^60^ as well as *S. aureus*^61^ without developing resistance^62^. Graphene-based nano-particles have been developed which kills gram-positive and negative bacteria upon near-infrared laser irradiation *via* the cross linking property^63^.

SlyD is a metallo-chaperone and the full-length protein can bind up to 7 Ni^2+^ ions with a histidine rich C-terminal extension of the PPIase and IF domain pair^64^. The *Ec*SlyD variant in the present study contains one Ni^2+^ binding site (His149, His151 and His153), which has been characterized by NMR spectroscopy^45^. The paramagnetic metal ion enhances the relaxation rates of NMR active nuclei in close proximity (paramagnetic relaxation enhancement (PRE) effect) resulting in a line broadening often below the detection limit. In the *Ec*SlyD/Ni^2+^ complex studied earlier, residues mainly following E141 at the C-terminus and residues close to Y34 in the PPIase domain show strong PRE^45^. An almost identical pattern was observed for the *Ec*SlyD complex with Cu^2+^ as the metal ion (Fig. S8c). The PRE pattern caused by the Cu^2+^ complex strongly differs from the pattern of single metal ions (Fig. 4a). Many more resonances are affected, which correspond to residues of the active sites of the PPIase and IF domains. As 9-anthracene methanol causes no significant changes in the NMR spectra of *Ec*SlyD (Figure S8a), we can conclude that only the Cu^2+^ coordination complex can effectively block the PPIase and chaperone function of *Ec*SlyD. We expect minor conformational changes of *Ec*SlyD to happen upon inhibitor binding, because none of the detectable resonances (Fig. 4a) show significant chemical shift changes. This is in agreement with the CD analysis of free and bound *Ec*SlyD (Figs 3a and S7).

*In vitro* studies of *Ec*SlyD substantiated that PPIase and chaperone function is located at two different domains and an interplay of both domains is required for full PPIase activity^19^,^20^. The isolated IF domain of *Thermos thermophilus* SlyD (*TtSlyD)* still has chaperone activity whereas the ΔIF variant lacks this function^65^. Excision of the IF domain from *Tt*SlyD led to a 100-fold decrease in PPIase activity towards protein substrates^65^ and a complete loss of activity for *Ec*SlyD^66^. The Cu^2+^ complex studied here binds *Ec*SlyD with a stoichiometry of 2:1 and inhibits both the chaperone and the PPIase activities (Fig. 2). NMR chemical shift mapping of complexed *Ec*SlyD (Fig. 4) revealed binding to both the active site of the PPIase domain^26^ and the IF domain^41^,^67^ with about the same affinity. The majority of mapped contacts are hydrophobic residues distant from the metal binding site at the C-terminus. Therefore, we propose that the anthracenyl terpyridine moiety of the Cu^2+^ complex facilitates binding of the metal not to the C-terminus of *Ec*SlyD but to both active sites by hydrophobic interactions to block its function.

In summary, it is expected that inhibition of cellular chaperone and PPIase activities will affect proper protein folding and functioning of their various substrates and thus bacterial proteostasis, which causes retardation of cell growth. Besides its chaperone and PPIase function, SlyD is known to be involved in other biochemical reactions, which are further cellular functions affected by SlyD inhibition. SlyD is present in both gram-positive and -negative bacteria. We demonstrated that both representatives of both families of pathogenic bacteria (*S. aureus* (gram-positive) and *P. aeruginosa* (gram-negative)) exhibited similar growth inhibition (IC_50_) as *E. coli* in the presence of the Cu^2+^ complex. Although we observed well documented binding to *Ec*SlyD and its functional consequences, we cannot rule out that the Cu^2+^ complex interacts with additional cellular proteins or DNA^68^ which cannot be completely revealed by the presented *in-cell* NMR experiment and need to be identified in future experiments. Targeting a molecular chaperone is a feasible way towards the generation of new antibacterial molecules, which is supported by our findings at a proof-of-principle stage.

## Methods

### Growth curve analysis

*E. coli* BL21(DE3) cells were grown in the double YT (dYT) media in a 50 ml of culture volume. For the growth curve analysis, a starting OD_600nm_ of 0.05 was taken by adding aliquots of the freshly grown culture at the log phase. Growth inhibition was analyzed by adding the 0–6 μM of the Cu^2+^ complex. These culture flasks were incubates at 37 °C with rotation at 300 rpm. Aliquots of growing culture were taken at different time point. Their growths were monitored by OD_ 600nm_. Aliquots were taken out until the OD _600nm_ of the control experiment without Cu^2+^ complex reached to the stationary phase.

In order to understand the role of SlyD in bacterial growth survival against the Cu^2+^ complex, *E. coli* BL21(DE3) cells were transformed with the *Ec*SlyD plasmid corresponding to the amino acid sequence (1–165). Growth curves in case of transformed bacteria were analyzed in the similar way as stated above. The *Ec*SlyD over production was initiated by inducing the culture with 1 mM of IPTG. The transformed *E. coli* BL21(DE3) cells were grown in presence of 30 μg/ml kanamycin.

In our experiment we have chosen SlyD (residues 1–165) of *E. coli*, a truncated version of full-length SlyD lacking the unstructured C terminal tail. Removing this tail does not affect the chaperone and PPIase functions of SlyD^21^,^26^. The Cu^2+^/Zn^2+^ complex or 9-anthracene methanol was dissolved in dimethyl sulfoxide (DMSO) and further diluted for the respective experiments. The final concentration of DMSO was <1% in any experiment.

### IC_50_ value determination

For the IC_50_ value determination, freshly grown *E. coli* BL21(DE3) cells or *Ec*SlyD transformed *E. coli* BL21(DE3) cells (non-induced with IPTG) at their log phase were used. The 10 ml of culture media was taken and the Cu^2+^ complex with concentrations ranging from 0–6 μM were added in a separate tube. A starting OD_600nm_ of 0.05 was used for this experiment. In a second experiment, *Ec*SlyD transformed cells were grown until OD_600nm_ of 0.6–0.7. This culture was then induced by adding 1 mM of IPTG. The culture was further grown for 3 h so that the cells had a sufficient number of *Ec*SlyD copies. These bacteria were used for a IC_50_ value determination as described for non-induced *E. coli* BL21(DE3) cells. All these cultures were incubated for 10 h at 37 °C with rotation at 300 rpm. OD_600nm_ of the bacteria without the Cu^2+^ complex was considered as 100% bacterial growth. The transformed *E. coli* BL21(DE3) cells were grown in presence of 30 μg/ml kanamycin. The experiments were performed in triplicate and repeated at least two times.

Pathogenic bacteria, *Staphylococcus aureus* and *Pseudomonas aeruginosa* were grown in nutrient broth. Their IC_50_ value was determined by co-incubating them with Cu^2+^ complex concentrations ranging from 0–8 μM. The growth of bacteria was monitored at OD_600nm_ as described above.

### In cell NMR

The *in cell* NMR experiment was performed as described earlier^69^. In brief *Ec*SlyD transformed *E. coli* BL21(DE3) cells were grown in M9 minimal media containing 30 μg/ml kanamycin until an OD_600nm_ of 0.6–0.7 was reached. These cells were induced with the 1 mM of IPTG and further incubated for 3 h at 37 °C with 300 rpm rotation. These cells were gently harvested by centrifugation at 5000 rpm at 4 °C. The cells were then washed 3 times with 50 mM Na_2_HPO_4_, 100 mM NaCl, pH 7.5. A 25% cells slurry with 10% D_2_O in a Shigemi tube was used in order to minimize the effect of settling of the bacteria. This culture was considered the control experiment. A 2D ^1^H-^15^N HSQC spectrum was recorded for 2 h at 25 °C. Thereafter, 100 μM of Cu^2+^ complex was added to this culture and incubated for 1 h at room temperature. After a short mixing period this slurry was transferred to the NMR tubes and a 2D ^1^H-^15^N HSQC spectrum was recorded for 2 h. In order to see if cell lysis might have occurred during the course of time and various treatments, the slurry was taken out of the NMR tube and centrifuged at 10000 rpm at 4 °C for 10 minutes. The supernatant was taken out and a 2D ^1^H-^15^N HSQC spectrum was recorded for 2 h.

### *Ec*SlyD expression and purification

For the expression and purification, the plasmid containing the gene corresponding to SlyD (1–165) of *E. coli* was transformed into the *E. coli* BL21(DE3) cells. The protein expression was carried out at 37 °C with IPTG induction. Purification of *Ec*SlyD was performed under denaturing conditions (GdmCl) as described earlier^22^.

### Molecular chaperone activity (insulin aggregation assay)

The insulin aggregation assay was performed as previously described^22^,^26^. In brief, insulin was dissolved in 6 M HCl and the pH was adjusted to 2.6 with NaOH. The aggregation assay was started at a concentration of 30 μM insulin (in 50 mM Na_2_HPO_4_, 100 mM NaCl, pH 7.5) with the addition of 10 mM DTT. Aggregation was retarded by addition of 40 μM *Ec*SlyD and the Cu^2+^ complex at concentrations of 0, 40, 70 and 100 μM. The reductive cleavage of the insulin chain B aggregation was monitored by increase in scattered light at 400 nm. The reaction was followed in a Jasco FP-6500 fluorescence spectrometer at 25 °C.

### PPIase activity

PPIase activity was analyzed as described earlier^22^. In brief, the catalytic efficiency of *Ec*SlyD was examined as a catalyst of the refolding of the reduced and carboxymethylated form of the S54G/P55N double mutant of ribonuclease T1 (RCM-T1). This reaction is limited by the slow *trans* to *cis* isomerization of Pro39. The reaction was carried out at 0–17 μM of RCM-T1 and a fixed concentration of 20 nM *Ec*SlyD. Enzyme inhibition was carried out at 0, 25, and 50 μM of Cu^2+^ complex. The experiments were carried in 50 mM Na_2_HPO_4_, 100 mM NaCl, pH 7.5 at 25 °C. The data was analysed by the Michaelis–Menten formalism with the enzyme kinetic module of Sigma Plot 12.

### Fluorescence binding

For the fluorescence studies, a solution of 5 μM of *Ec*SlyD was used for binding studies. In a typical titration experiment, 4 μM of Cu^2+^ complex was added at each step and the spectrum was recorded after a short mixing period. Titration of Cu^2+^ perchlorate or 9-anthracene methanol was also carried out in the same way. A blank titration (without protein) was recorded, where the Cu^2+^ complex or Cu^2+^ perchlorate or 9-anthracene methanol was added. This blank titration was subtracted from the main experiment before the analysis. The titration experiment was carried in 50 mM Na_2_HPO_4_, 100 mM NaCl, pH 7.5. The whole solution was excited at 280 nm on a Jasco FP-6500 fluorescence spectrometer at a temperature of 25 °C.

Fluorescence data were analyzed according to the following equation:


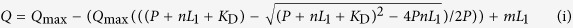


with *P* = (*P*_0_/(*V*_0_ + *L*))*V*_0_ and *L*_1_ = (*L*_0_/(*V*_0_ + *L*))*L* and *Q*–fluorescence intensity, *P*–protein concentration, *L*–ligand concentration, *V*–volume, *n*–binding sites and 0–indicates start point.

### CD spectroscopy

For the CD spectroscopic studies a solution of 10 μM of *Ec*SlyD was used. In typical titration experiments 10 μM of the Cu^2+^ complex was added. After a short mixing period, a spectrum was recorded after each titration step. Titration of Cu^2+^ perchlorate or 9-anthracene methanol was carried out at the same protein concentration. Cu^2+^ complex or Cu^2+^ perchlorate or 9-anthracene methanol titrated against buffer was used as blank. This blank titration was subtracted from the main experiment before the analysis. The titration experiment was carried in 50 mM Na_2_HPO_4_, 100 mM NaCl, pH 7.5. The spectrum was recorded on Jasco J815 spectrometer at a temperature of 25 °C.

### *In vitro* NMR spectroscopy

The spectra were recorded at a concentration of 60 μM of *Ec*SlyD. NMR titration experiments were carried out by the addition of 1 μl of 10 mM Cu^2+^ complex until there was no further change in NMR intensity observed. The spectrum corresponding to the 1:2 complex formations was used for the analysis. The NMR titration with Cu^2+^ perchlorate or 9-anthracene methanol was carried out in the same way. The spectra were recorded in 50 mM Na_2_HPO_4_, 100 mM NaCl, pH 7.5, with a Bruker 800 MHz Avance III spectrometer equipped with a CP–TCI cryoprobe at 25 °C. Spectra were processed using the programs NMRPipe and NMR Draw. The previously determined NMR assignments of the *Ec*SlyD resonances determined previously were used for analysis^26^.

## Additional Information

**How to cite this article:** Kumar, A. and Balbach, J. Targeting the molecular chaperone SlyD to inhibit bacterial growth with a small molecule. *Sci. Rep.* **7**, 42141; doi: 10.1038/srep42141 (2017).

**Publisher's note:** Springer Nature remains neutral with regard to jurisdictional claims in published maps and institutional affiliations.

## Supplementary Material

Supplementary Information
