# Supplementary material for: Targeting the molecular chaperone SlyD to inhibit bacterial growth with a small molecule
Source: Sci Rep. 2017 Feb 8;7:42141. doi: 10.1038/srep42141 (PMC5296862; doi:10.1038/srep42141)
Supplement: Supplementary Information [file srep42141-s1.pdf]

Supplementary information

Targeting the molecular chaperone SlyD to inhibit bacterial growth with a small molecule

Amit Kumar[1,2]* *and Jochen Balbach*[2,3,]*

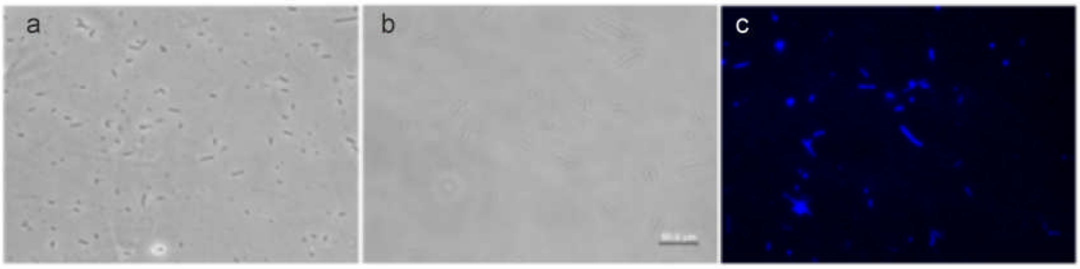

Figure S1: Morphological analysis of the *Escherichia coli* strain BL21(DE3) in the presence and absence of the Cu^{2+} complex. (A) Phase contrast image of untreated control bacteria. (B) Phase contrast image of Cu^{2+} complex treated bacteria. (C) Fluorescence microscopic image of (B). The image was taken after exciting the bacteria using the UV light provided with the Olympus microscope BX51.

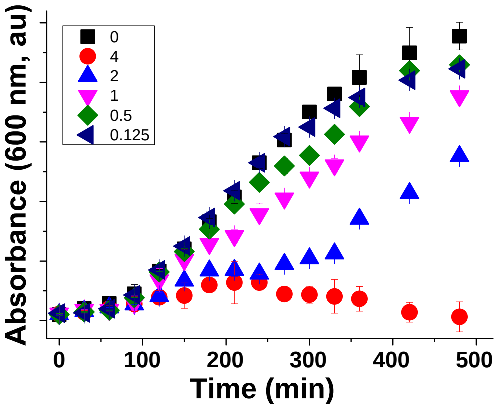

Figure S2: Growth curve analysis of the *E. coli* strain BL21(DE3). Values in the inset indicated the tested concentrations of the Cu^{2+} complex. Error bars correspond to two independent experiments run in triplicate.

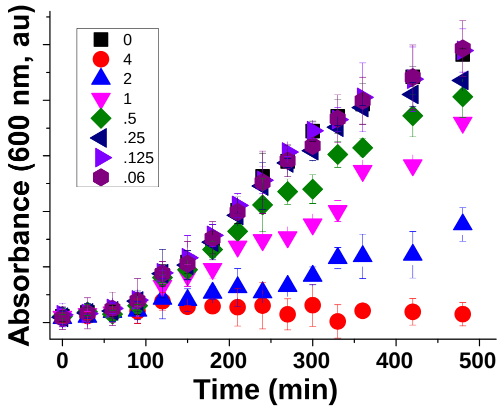

Figure S3: Growth curve analysis of the *E. coli* strain BL21(DE3) transformed with a plasmid containing gene for *Ec*SlyD in the presence and absence of Cu^{2+} complex at the denoted concentrations. Error bars correspond to two independent experiments run in triplicates.

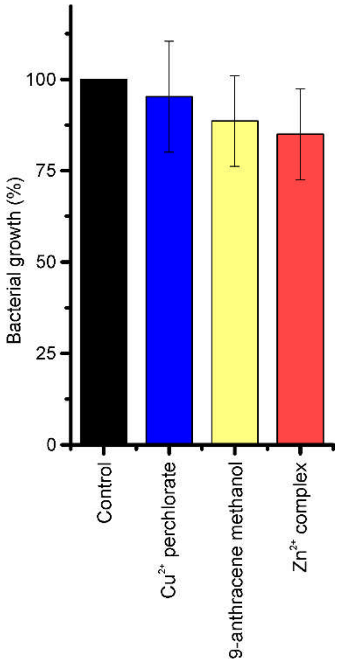

Figure S4: Control experiments of bacterial growth of BL21(DE3) cells. The experiments were performed at 4 µM of the respective inhibitor and errors result from two independent experiments run in triplicates.

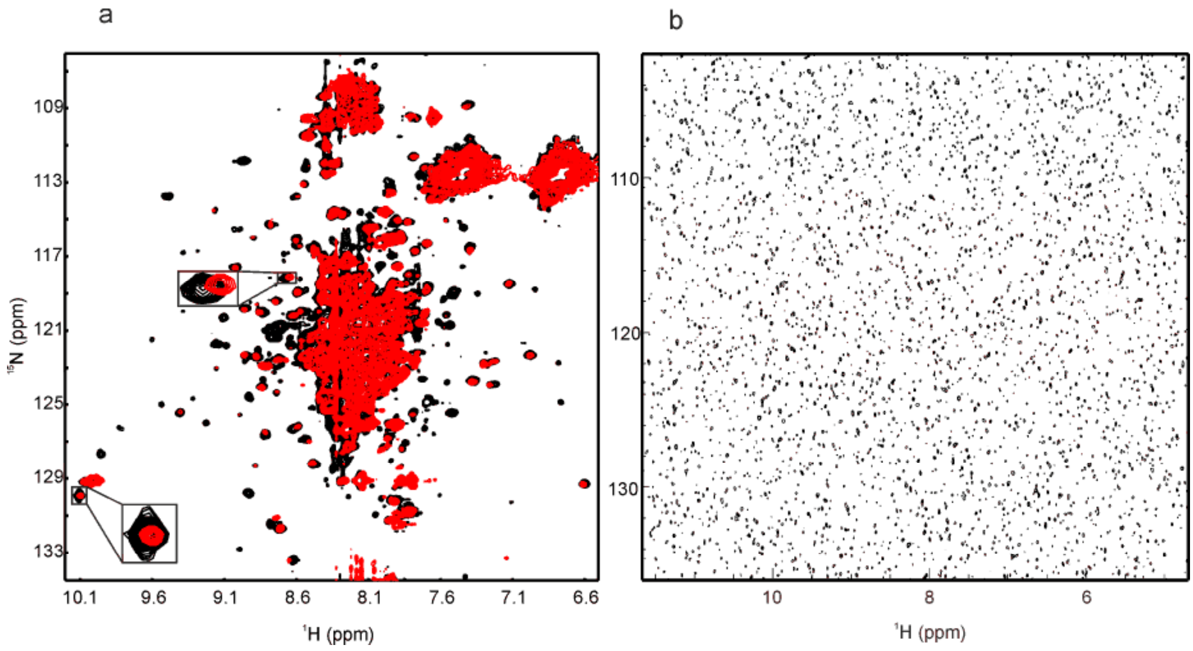

Figure S5: *In cell* NMR spectra of *Ec*SlyD. (A) Overlaid 2D ^{1}H-^{15}N HSQC spectra are shown before (black) and after addition of the Cu^{2+} complex (red). A section of these spectra are shown in Figure 2A. (B) 2D ^{1}H-^{15}N HSQC of supernatant indicating no NMR detectable cell lysis during the course of experiments.

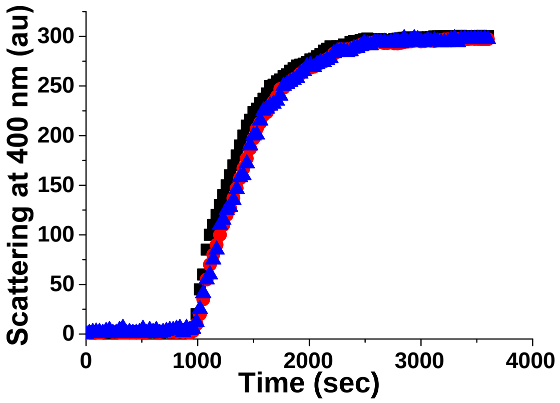

Figure S6: Molecular chaperon activity analysed by insulin chain B aggregation assay in the presence of 100 μM control molecules: ■ – in presence of Cu^{2+} perchlorate, ● – in presence of 9-anthracene methanol and ▲ – in presence of Zn^{2+} complex.

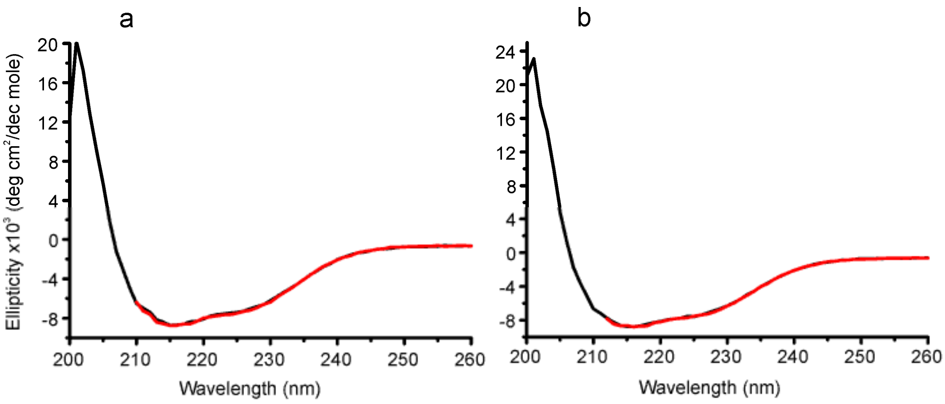

Figure S7: CD spectra of *Ec*SlyD in presence and absence of 50 μM control molecules. The black spectrum corresponds to free *Ec*SlyD and red in presence of. (A) Cu^{2+} perchlorate and (B) 9-anthracene methanol. High absorption beyond 215 nm did not allow useful spectra to be recorded upon addition of these molecules.

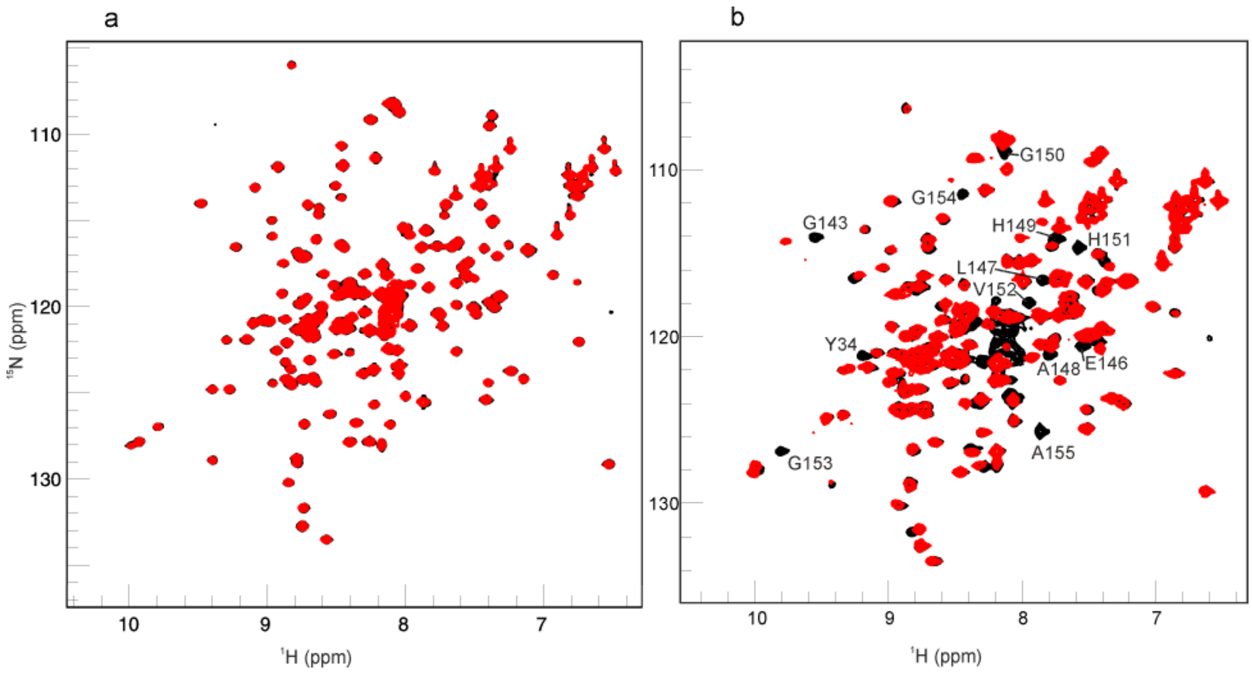

Figure S8: Superposition of 2D ^{1}H-^{15}N HSQC spectra of ^{15}N *Ec*SlyD in the free-state (black) and bound to (A) 9-anthracene methanol and (B) Cu^{2+} perchlorate. Residues with strongly reduced cross peak intensities are labelled.

Residues at FKBP domain

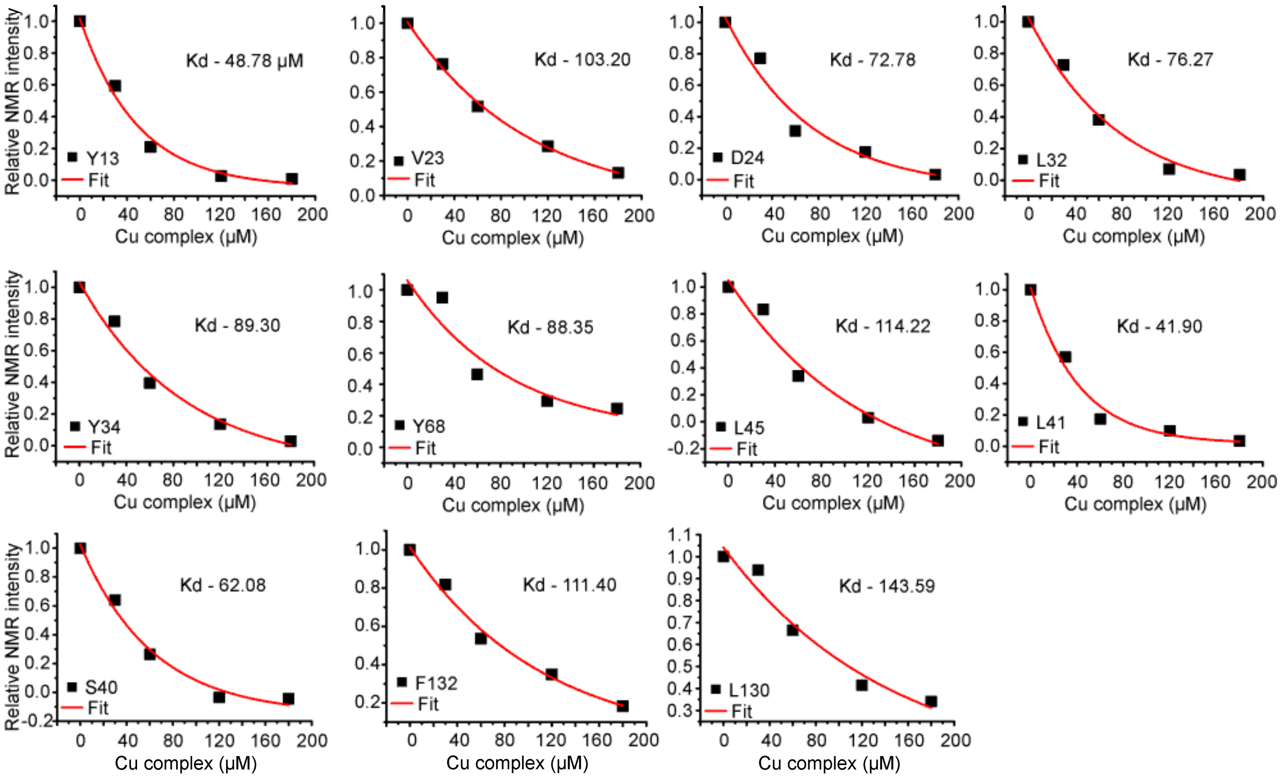

Residues at IF domain

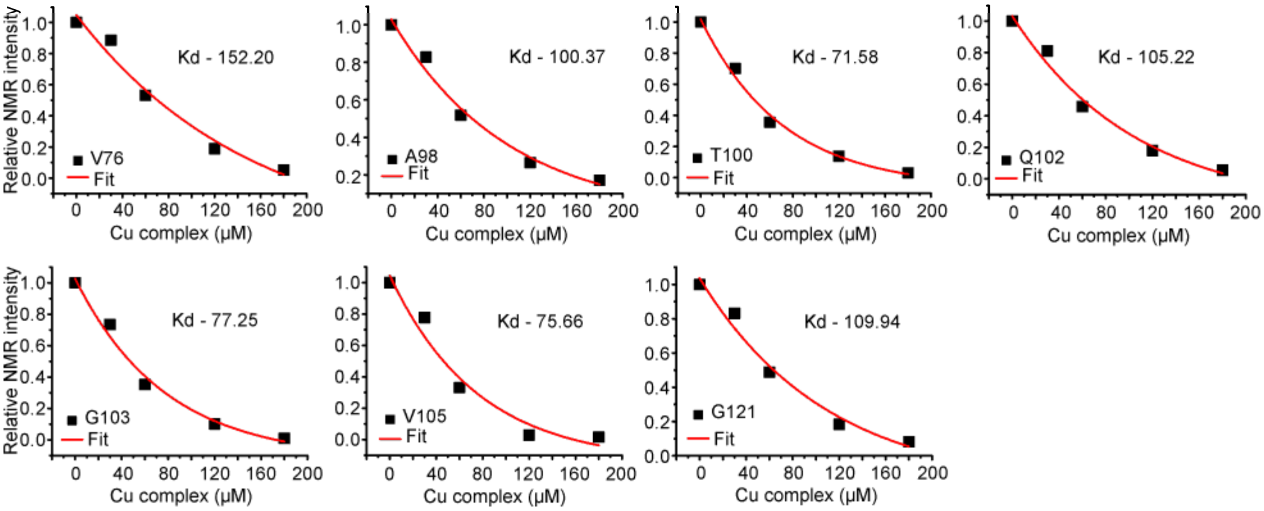

Figure S9: Residues specific binding analysis based on different amino acids at the FKBP (top) and IF domain (bottom). The NMR data were fitted according to the Eq (i) given in the experimental section.

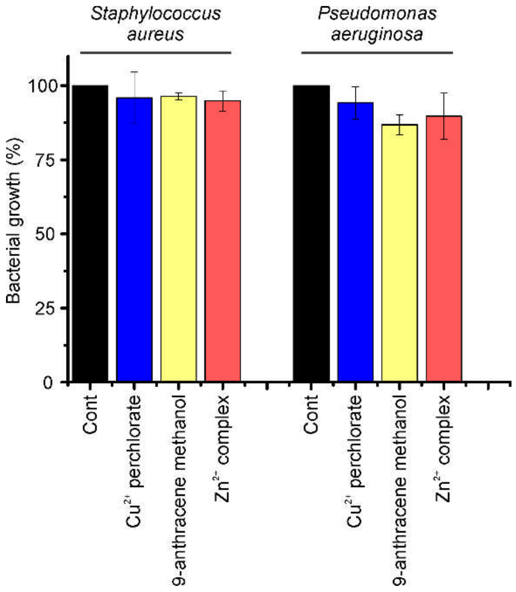

Figure S10: Control experiments of cell growth in the case of pathogenic bacteria. The experiments were performed at a concentration of 8 µM of the indicated molecules and errors result from two independent experiments run in triplicate.
